# Circulating microRNAs correlate to clinical parameters in individuals with allergic and non-allergic asthma

**DOI:** 10.1186/s12931-020-01351-x

**Published:** 2020-05-07

**Authors:** Julie Weidner, Linda Ekerljung, Carina Malmhäll, Nicolae Miron, Madeleine Rådinger

**Affiliations:** 1 Krefting Research Centre, Sahlgrenska Academy, University of Gothenburg, Gothenburg, Sweden; 2 Clinical Immunology, Sahlgrenska University Hospital, Gothenburg, Sweden

**Keywords:** Asthma, microRNAs, qPCR, Serum

## Abstract

**Background:**

Asthma is a chronic airway disease affecting millions of people. Better methods to define asthma subgroups using clinical parameters and molecular biomarkers are crucial in the development of personalized medicine.

**Objective:**

The aim of this study was to determine if circulating microRNAs (miRNAs) may be used to distinguish well–defined asthma groups.

**Methods:**

Blood serum from 116 well-defined subjects, including healthy controls and individuals with allergic or non-allergic asthma, from the West Sweden Asthma Study were included. Serum was analyzed for circulating miRNA expression of miR-126, − 145, −146a, − 155, − 223, and -374a and eosinophil cationic protein (ECP). Correlations between clinical characteristics and circulating miRNA expression as well as potential miRNA gene targets were investigated.

**Results:**

A subset of miRNAs were differentially expressed between allergic and non-allergic asthmatic individuals. Alterations in expression of miR-155, −146a, −374a and − 145 were observed in allergic asthmatics in response to inhaled corticosteroid usage. Additionally, miR-223 and miR-374a expression varied in non-allergic asthmatics based on blood eosinophil numbers. Numerous clinical parameters, including lung function measurements, correlated with subsets of miRNAs. Finally, pathway analysis revealed a potential role for inhaled corticosteroid induced miRNAs in leukocyte regulation, IL-6 signaling and glucocorticoid response.

**Conclusion:**

Circulating miRNA expression was altered in subjects with allergic and non-allergic asthma and correlated to clinical parameters including lung function and potential gene targets involved in immune processes. This combination of clinical and molecular data may be a basis for the further, more precise classification of asthma subgroups. Taken together, these findings would further asthma research and benefit future patients through the discovery of molecular mechanisms as well as identifying asthma subgroups contributing to the development of personalized medicine.

## Introduction

Asthma is a heterogeneous disease affecting more than 300 million people worldwide and causing substantial socioeconomic burden [1]. The disease is characterized by bronchoconstriction and hyper-responsiveness of the airways, but many of the molecular mechanisms causing specific asthma subtypes remains unknown. Currently, most asthmatics are described based on atopy or prevalence of cell type, such as eosinophils or neutrophils. In addition to the clinical phenotypes, there is an overwhelming need for a better understanding of the molecular characteristics and distinct pathophysiological mechanisms making up individual subgroups of asthma, thus defining asthma endotypes [2]. Due to the heterogeneity of the disease, it is naïve to believe that one treatment fits to all asthma subgroups, therefore, personalized profiling and treatment is needed.

Recently, non-coding RNAs have been shown to be prevalent regulatory molecules involved in a wide array of diseases and cellular processes [3–8]. microRNAs (miRNAs) are small, 18–24 nucleotide, RNAs responsible for the regulation of gene expression mostly through the binding and down-regulation of target mRNAs. Briefly, miRNAs are transcribed in the nucleus before export to the cytoplasm, processing, and loading of the final, mature miRNA into the Argonaute protein. This process has been thoroughly described in detail in several recent reviews [6, 7, 9]. Although miRNAs have been extensively studied in cancer [4, 5], their role in asthma remains relatively unknown. Mouse models of airway hyper-responsiveness and inflammation have elucidated potential mechanisms for miRNAs in the airways, but relatively few studies have been conducted in human subjects. Most recently, large scale screens using RNA-sequencing platforms or microarrays have identified potential miRNAs that may be associated with childhood asthma or allergic diseases such as allergic rhinitis [10–14]. Furthermore, miRNAs are ubiquitous throughout the body, both within the cell and in a variety of bodily fluids such as serum, plasma, breast milk and urine [15–19]. Due to the stable nature of miRNAs in fluids that can be acquired non-invasively, they are ideal candidates for biomarkers and potential therapeutic targets.

We set out to examine if a set of miRNAs could be monitored in serum and used as circulating non-invasive biomarkers to distinguish various asthma subgroups. Six miRNAs were chosen for this study based on their previous identification in murine models of asthma as well as their observed involvement in human immune responses [20–25]. Importantly, these miRNAs have mainly been examined in models of allergic asthma [4, 21, 24–33], thus, we wanted to examine if expression would be altered in individuals with non-allergic asthma. We found that the examined miRNAs showed distinct expression patterns in allergic and non-allergic asthma subgroups. Furthermore, the expression of a subset of miRNAs was significantly different depending on inhaled corticosteroid (ICS) usage or blood eosinophil level. Circulating eosinophil levels correlated to levels of eosinophil cationic protein (ECP) in the blood. Several of the circulating miRNAs correlated to a variety of clinical characteristics, including lung function parameters. Finally, in silico pathway analysis revealed that miRNAs increased under ICS usage were observed to target genes involved in leukocyte regulation and glucocorticoid response. Taken together, we believe that these miRNAs form a distinct circulating profile aiding in distinguishing different asthma subgroups. These profiles may be useful in the development of future biomarkers and be used as additional criteria in the development of asthma endotypes.

## Materials and methods

### Study subject criteria

Subjects were selected from the West Sweden Asthma Study (WSAS [34]). All subjects were free of systemic inflammatory diseases and cancers and, in addition, healthy subjects were free of lung diseases. All asthma was physician diagnosed and no other lung diseases were present. Individuals with asthma were of mild-moderate disease severity as determined by GINA guidelines [1] at the time of recruitment. Asthma was diagnosed from reports of common symptoms, use of asthma medication, or a provocation dose (PD20) for methacholine below a cumulative dose of 1.94 μg or a FEV_1_ reversibility greater than 12%. Exclusion criteria for this group included being diagnosed by a physician as having emphysema, chronic obstructive pulmonary disease or chronic bronchitis. Spirometry, differential cell counts, serum IgE and/or skin prick test (SPT) were performed on all individuals. The SPT used a standard panel of 11 inhalant allergens composed of birch, mugwort, timothy, horse, dog, cat, cockroach, *Cladosporium, Alternaria, Dermatophagoides farinae and D. pteronyssinus* (ALK, Hørsholm, Denmark). The SPT test was considered positive with a wheal and flare reaction ≥3 mm for at least one allergen. Routine differential cell counts from whole blood were analyzed by the Clinical Chemistry Laboratory (Sahlgrenska University Hospital, Gothenburg, Sweden). Asthmatics were considered to have high numbers of eosinophils if blood eosinophils were ≥ 0.4 × 10^9^/L and low numbers of eosinophils if values were ≤ 0.1 × 10^9^/L. Control subjects did not report asthma symptoms, were non-reactive to methacholine or non-reversible, were SPT negative and had a low number of blood eosinophils. All groups were non-smokers or former smokers with smoking cessation of at least 5 years and a maximum of 10 pack years. For the serum miRNA analysis, 116 individuals (24 healthy and 92 asthma) were included and further divided based on allergy status, circulating eosinophil level and ICS usage (Tables 1 and 2). This study was approved by the ethics committee at Gothenburg University (DNR 593–08) and all subjects provided written informed consent.

**Table 1:**
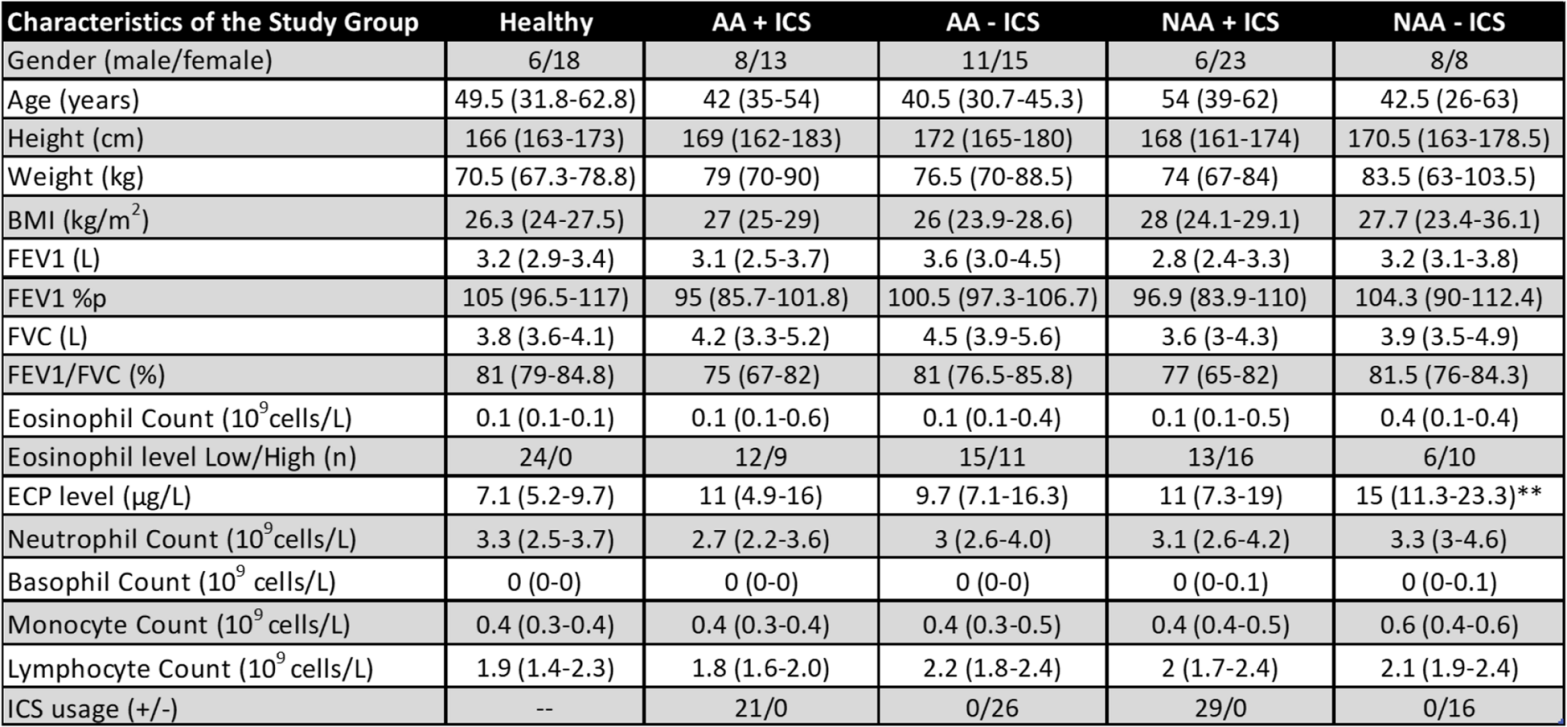
Characteristics of the study group divided based on circulating eosinophil levels

Data is presented as median with 25–75% in parenthesis. * = versus Healthy; # = versus AA Eos high; § = versus NAA Eos high. *p* < 0.05 *, *p* < 0.01 **, *p* < 0.001 = ***; *p* < 0.0001 = ****. Eosinophil data is shown as median of total subjects with the range divided into Low (LE) and High (HE) groups and number of subjects in each low/high group are given. *AA* Allergic Asthma. *NAA* Non-allergic asthma. *%p* percent predicted. *FEV1* Forced Expiratory Volume in 1 s. *FVC* Forced Vital Capacity, *BMI* Body Mass Index, *ICS* Inhaled Corticosteroids

**Table 2:**
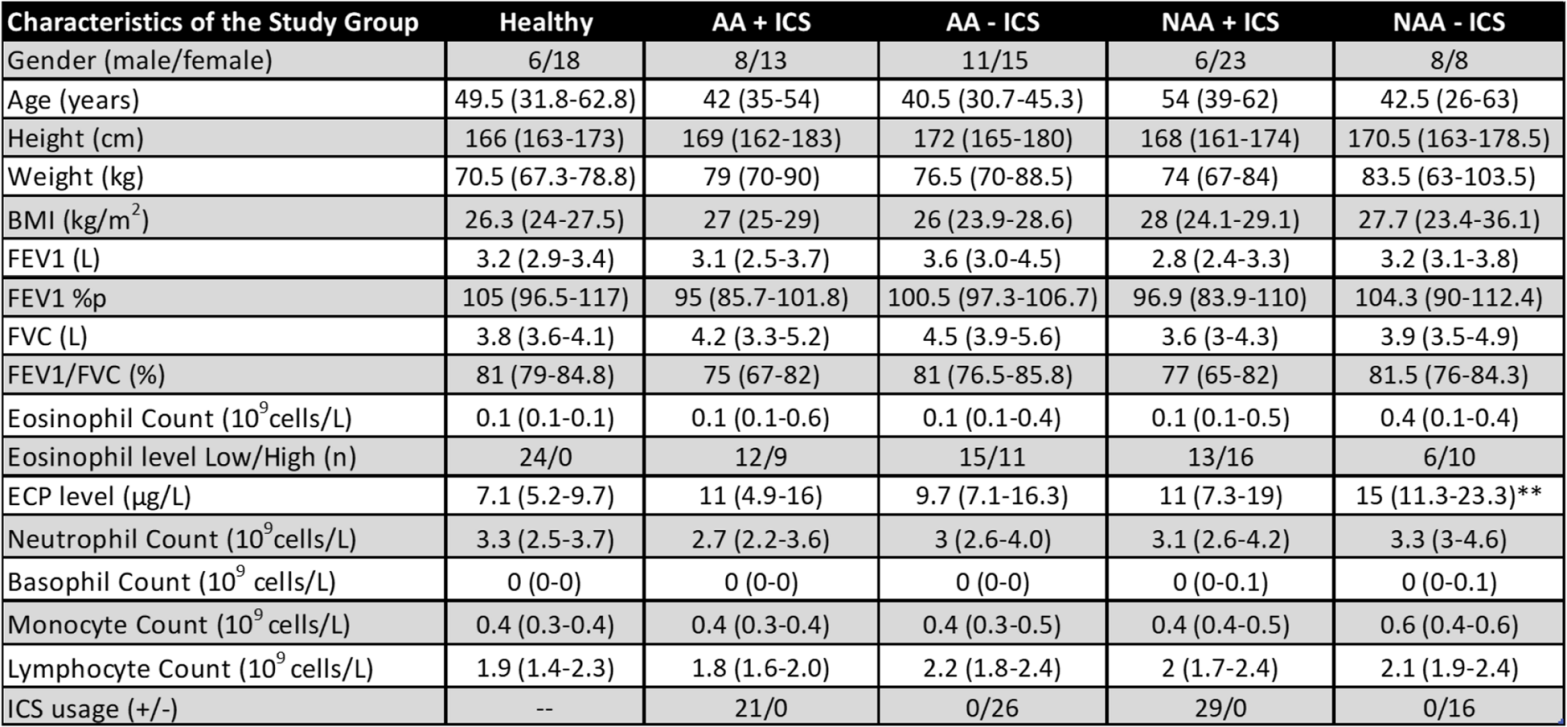
Characteristics of the study group divided based on inhaled corticosteroid usage

Data is presented as median with 25–75% in parenthesis. * = versus Healthy; *p* < 0.05 *, *p* < 0.01 **. Eosinophil data is shown as median of total subjects with the range divided into Low (LE) and High (HE) groups and number of subjects in each low/high group are given. *AA* Allergic Asthma. *NAA* Non-allergic asthma. %p = percent predicted. *FEV1* Forced Expiratory Volume in 1 s. *FVC* Forced Vital Capacity. *BMI* Body Mass Index. *ICS* Inhaled Corticosteroids

### RNA isolation

Before isolation, frozen serum aliquots were thawed and centrifuged for 10 min at 20 000 x g to clear the serum. Qiazol (Qiagen, Hilden, Germany) was added to 140 μl cleared serum in a Phasemaker tube (Invitrogen, California, USA) and total RNA was isolated using the Qiagen Serum/plasma miRNeasy micro kit according to manufacturer’s instructions (Qiagen) and the minimum recommended volume (14 μl) was used for eluting the sample. RNA concentration was determined by chip-based capillary electrophoresis (Agilent RNA 6000 Pico RNA Kit and Aglient 2100 Bioanalyzer, Agilent Technologies, Waldbronn, Germany). For the reverse transcription reaction, 2 μl of total RNA was reverse transcribed into cDNA for miRNA analysis using the miRCury LNA RT kit (Qiagen). UniSp6 RNA Spike-In was added into each reaction as a control in the cDNA synthesis step.

### qPCR

All qPCR analysis was performed on a Bio-Rad CFX96 real time PCR detection system(Hercules, CA, USA). For miRNA qPCR analysis, reactions were set up according to the manufacturer’s instructions using miRCury LNA primers and miRCury SYBR green (Qiagen). miR-103a-3p was used as a reference miRNA and expression is depicted as 2^-dCT^. Pre-designed miRCury LNA primers used were the following: miR-126 (MIMAT0000445), miR-145 (MIMAT0000437), miR-146a (MIMAT0000449), miR-155 (MIMAT0000646), miR-223 (MIMAT0004570) and miR-374a (MIMAT0000727).

### Eosinophil cationic protein analysis

Serum ECP was measured with a fluoroenzyme immunoassay (ImmunoCAP™ ECP, Phadia AB, Thermo Fisher Scientific, Uppsala, Sweden) on a Phadia 250 instrument according to the manufacturer’s instructions. Samples were undiluted and special attention was paid to specimen collection and preparation. Blood samples were kept at room temperature (20–24 °C, 60–120 min) before centrifuging and separating the serum. Serum aliquots were stored at − 80 °C before analysis.

### Statistics

Descriptive statistics are presented as median with each individual represented by a single point. miRNA expression was analyzed using Kruskal-Wallis (KW) test followed by Dunn’s multiple comparison post-test (D). *P* < 0.05 was considered significant. Spearman correlation testing was performed to determine correlations between miRNA expression and clinical characteristics in all subjects (*n* = 116). A multiple logistic regression was performed on asthma subjects to determine if miRNAs were able to distinguish between study groups (Supp Fig. 3). Statistical analyses were performed using Graph Pad Prism 8 (Graph pad software Inc., San Diego, USA).

### Pathway analysis

Putative gene targets for each miRNA of interest were obtained from miRDB [35], miRNet [36] and miRTarBase [37] and a list of common targets was compiled. The common target lists for the individual miRNAs were then compared to one another to attain a list of 98 shared gene targets using Venny [38]. In order to determine interactions between shared targets, the list was examined via String [39]. The minimum required interaction score was set to highest confidence interactions (0.9), all possible active interaction sources were allowed, the network edges were set to display the predicted molecular mode of action and unconnected nodes were allowed to be displayed. Functional enrichments including Biological Processes (Gene Ontology, GO), KEGG (Kyoto encyclopedia of genes and genomes) pathway analysis and Reactome pathways were analyzed (Supp Tables 1-3).

## Results

### Circulating miRNAs show altered expression in asthma groups

**Fig. 1:**
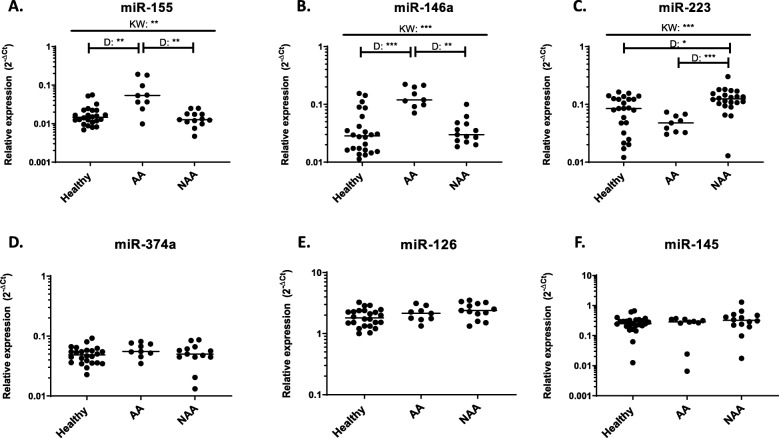
Circulating miRNAs exhibit alterations in expression in asthma subgroups. Expression of six candidate miRNAs (**a-f**) were examined in blood serum from individuals with asthma on inhaled corticosteroids and with eosinophilia. Allergic asthmatic individuals (AA) and non-allergic asthmatics (NAA) had high levels of circulating eosinophils (> 0.4 × 10^9 cells/L) and healthy subjects had low levels of circulating eosinophils (< 0.1 × 10^9 cells/L). Each dot is equivalent to one subject. Healthy = 24, AA = 9, NAA = 13. Expression is shown as 2^-dCt. Kruskal-Wallis followed by Dunns post-test was performed. *p* < 0.05 = *; *p* < 0.01 = **; *p* < 0.001 = ***

As miRNAs are extremely stable in bodily fluids [15–19] we set out to determine if differences could be observed amongst healthy, allergic (AA) and non-allergic asthmatic (NAA) subjects. We began by examining miRNAs in a small pilot asthma cohort (AA = 9, NAA = 13) to determine if we observed any expression alterations. We selected healthy subjects with low eosinophil levels (< 0.1 × 10^9^ cells per L) and AA and NAAs with high circulating eosinophil levels (> 0.4 × 10^9^ cells per L) and on ICS (Table 1). We found that both miR-155 and miR-146a exhibited significantly increased expression in AA asthmatics compared to healthy or NAA subjects (Fig. 1a & b). miR-223 was also altered in the asthma groups with expression in NAAs being significantly higher than healthy or AAs (Fig. 1c). Three other miRNAs, miR-126, −374a and − 145 all exhibited slight, but insignificant changes between groups examined (Fig. 1d-f).

### Inhaled corticosteroid usage affects circulating miRNA expression independently of eosinophil high status

**Fig. 2:**
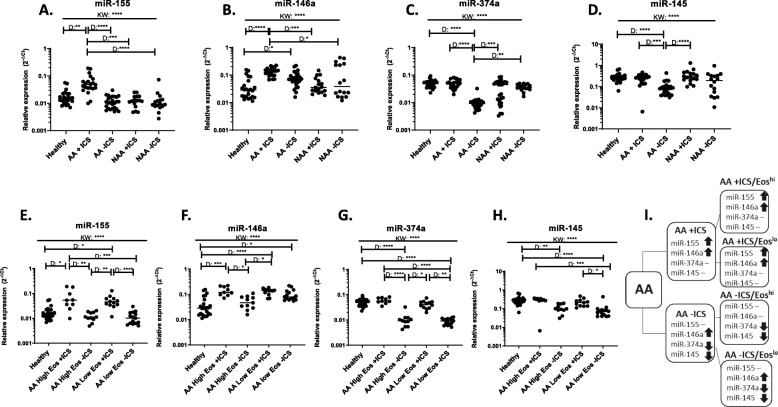
Presence of inhaled corticosteroids affects miRNA expression in allergic asthmatics. Asthma subgroups were divided into individuals using inhaled corticosteroids (ICS) or steroid naïve in (**a-d**) or, additionally, by circulating eosinophil levels (**e-h**) where High Eos are > 0.4 × 10^9 cells/L and healthy subjects and Low Eos individuals had < 0.1 × 10^9 cells/L. (**i**) A flow chart to describe miRNA expression for each group is shown. ↑ indicates increased expression compared to healthy, ↓ indicates decreased expression compared to healthy and – indicated no change from healthy. Each dot is equivalent to one subject. Healthy = 24, AA+ICS = 21, AA-ICS = 26, NAA + ICS = 29, NAA-ICS = 16, AA High Eos + ICS = 9, AA Low Eos + ICS = 12, AA High Eos-ICS = 11, AA Low Eos-ICS = 15. Expression is shown as 2^-dCt. Kruskal-Wallis followed by Dunns post-test was performed. *p* < 0.05 = *; *p* < 0.01 = **; *p* < 0.001 = ***; *p* < 0.0001 = ****

As our initial pilot asthma subjects were all on ICS and had high levels of circulating eosinophils (> 0.4 × 10^9^ cells per L) despite ICS treatment, we questioned whether either factor may affect miRNA expression. To determine the effect of ICS on individuals with asthma, we added subjects that were ICS naïve (Fig. 2; Table 2). We found that several miRNAs exhibited altered expression in AAs who were on (+) or off (−) ICS. miR-155 was significantly increased in AA+ICS subjects whereas AA-ICS subjects exhibited expression similar to healthy individuals (Fig. 2a). Similar to miR-155, miR-146a was increased in AA+ICS individuals. Additionally, an increase in miR-146a expression compared to healthy subjects was also observed in AA-ICS individuals, but not to the same extent as was seen in AA+ICS subjects (Fig. 2b). Moreover, miR-374a and − 145 exhibited significant decreases in expression in AA-ICS (Fig. 2c&d). No significant differences were observed in any miRNA in NAAs + or - ICS compared to healthy individuals (Fig. 2a-d).

We next wanted to determine if circulating eosinophil numbers had an effect on altered miRNA expression in the presence of ICS. AA individuals with differing eosinophil levels were selected and divided into high circulating eosinophil (> 0.4 × 10^9^ cells per L) and a low eosinophil levels (< 0.1 × 10^9^ cells per L) both + and - ICS (Fig. 2e-h). The significant increase in miR-155 or miR-146a expression was not altered based on circulating eosinophil levels in AA+ICS individuals compared to healthy subjects (Fig. 2e&f). Interestingly, miR-146a levels were increased in AA-ICS with low circulating eosinophil numbers, but not those with high levels. Additionally, the decrease observed in miR-374a or miR-145 in AA-ICS subjects was similar in both high and low eosinophil individuals (Fig. 2g&h). Regardless of circulating eosinophil number, ICS status dictated the significant increase in miR-155 expression in AA+ICS individuals and decreased trend in miR-374a and miR-145 in AA-ICS individuals (Fig. 2i).

### Circulating eosinophil levels alters the expression of miR-223 and miR-374a in NAAs

**Fig. 3:**
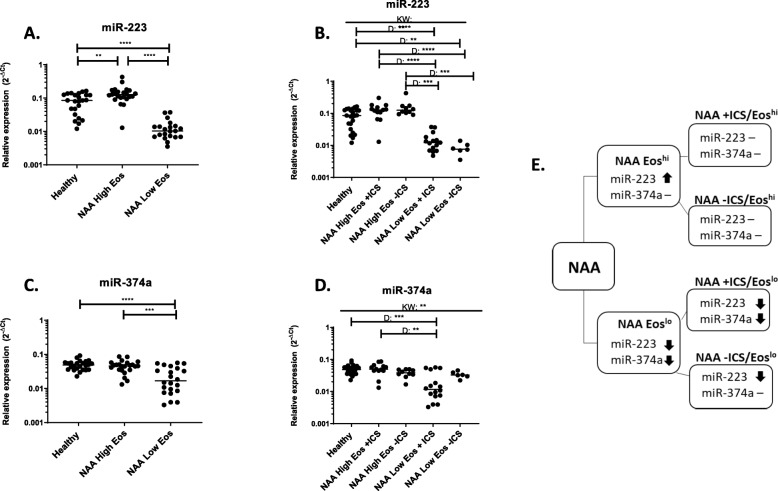
Expression of miR-223 and miR-374a correlate to eosinophil levels in non-allergic asthmatics. Expression of two circulating miRNAs were observed to change based on eosinophil numbers. Individuals with non-allergic asthma were divided based on circulating eosinophil cell level- where High Eos are > 0.4 × 10^9 cells/L and healthy subjects and Low Eos individuals had < 0.1 × 10^9 cells/L (**a** and **c**) or eosinophil level and ICS usage (**b** and **d**). (**e**) A flow chart to describe miRNA expression for each group is shown. ↑ indicates increased expression compared to healthy, ↑ indicates increased expression compared to healthy, ↓ indicates decreased expression compared to healthy and – indicated no change from healthy. Each dot is equivalent to one subject. Healthy = 24, NAA High Eos = 23, NAA Low Eos = 22, NAA High Eos + ICS = 13, NAA High Eos-ICS = 10, NAA Low Eos + ICS = 16, NAA Low Eos-ICS = 6. Expression is shown as 2^-dCt. Kruskal-Wallis followed by Dunns post-test was performed. *p* < 0.05 = *; *p* < 0.01 = **; *p* < 0.001 = ***; *p* < 0.0001 = ****

Although the most significant increases in miRNA expression were seen in AAs, there was a slight, but significant increase in miR-223 in NAAs (Fig. 1c). Thus, we wanted to determine if circulating eosinophil levels or ICS status in NAAs may influence this increased expression. We found that miR-223 expression was increased in individuals with high circulating eosinophil levels and significantly decreased in individuals with low circulating eosinophil levels as compared to healthy individuals (Fig. 3a). Similarly, we found that NAA low eosinophil individuals also exhibited significantly decreased expression of miR-374a compared to healthy or NAA high eosinophil individuals (Fig. 3c). When NAA groups were divided by ICS status and eosinophil numbers, it became clear that miR-223 expression was altered by circulating eosinophil number, but not ICS usage (Fig. 3b). Similar results were observed for miR-374a expression with no significant differences seen among those with high eosinophil numbers regardless of ICS status, but the most significant change was seen in NAA low eosinophil individuals on ICS (Fig. 3d). Interestingly, NAA-ICS low eosinophil individuals were not as low in miR-374a expression as their counterparts on ICS. Overall, the expression changes observed in miR-223 and miR-374a in NAA individuals appeared linked to circulating eosinophil levels (Fig. 3e).

### Circulating miRNAs correlated to clinical parameters

**Fig. 4:**
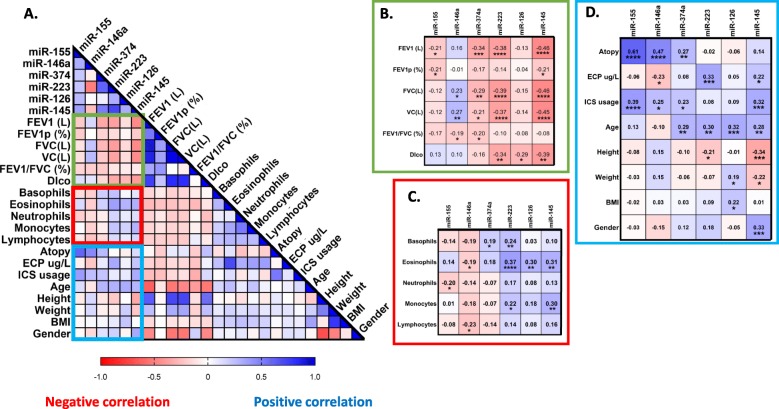
Serum miRNAs correlate to clinical parameters. Spearman correlations were performed between circulating miRNA expression and various clinical parameters in (**a**) (*n* = 116). FEV1 = Forced Expiratory Volume in 1 s. FVC = Forced Vital Capacity. p (%) = percent predicted. Dlco = Diffusion capacity of the lung for carbon monoxide. BMI = Body Mass Index. ICS = Inhaled Corticosteroids. ECP = Eosinophil cationic protein. Cells are 10^9/L, height in cm, weight in kg. Each boxed section (**b-d**) is a cut out and enlarged section highlighted from the larger correlation matrix (**a**) and contains the correlation coefficient for each pair and significant correlations (*) if present. *p* < 0.05 = *; *p* < 0.01 = **; *p* < 0.001 = ***; *p* < 0.0001 = ****

As we have extensive characterization of the subjects used in our study, we next looked for correlations with parameters clinically relevant to asthma and our miRNAs (Fig. 4a). When all subjects were taken into account, we found that several miRNAs negatively correlated to lung function parameters such as forced expiratory volume in 1 s (FEV1) and forced vital capacity (FVC) with miR-145 showing the strongest correlation followed by miR-223 and miR-374a, FEV1/FVC (miR-146a and miR-374a) and diffusion capacity for carbon monoxide (Dlco; miR-145, − 223 and − 126; Fig. 4b) in a similar significant manner. miR-223 showed the highest positive correlation to circulating eosinophil number (Fig. 4c). miR-155, −146a and -374a were significantly associated with atopy. Additionally, we found that miR-155, −146a, −374a and miR-145 all positively correlated with ICS usage. Interestingly, four of the miRNAs examined correlated with varying degrees to age (miR-374a, − 223, − 126 and − 145; Fig. 4d). miR-145 showed significant correlations to height, weight and gender (Fig. 4d). ECP significantly correlated with miR-223 and miR-145 in a positive manner and negatively correlated with miR-146a, similar to what was observed with correlations to eosinophil numbers. To determine if our candidate miRNAs may be feasible as potential future biomarkers, we performed a multivariate logistical regression analysis on our asthma cohort. Receiver operating characteristics showed the highest area under the curve for miR-146a (0.95), miR-223 (0.93) and miR-155 (0.90) in distinguishing between NAA and AA subjects (Supp Fig. 3).

**Fig. 5:**
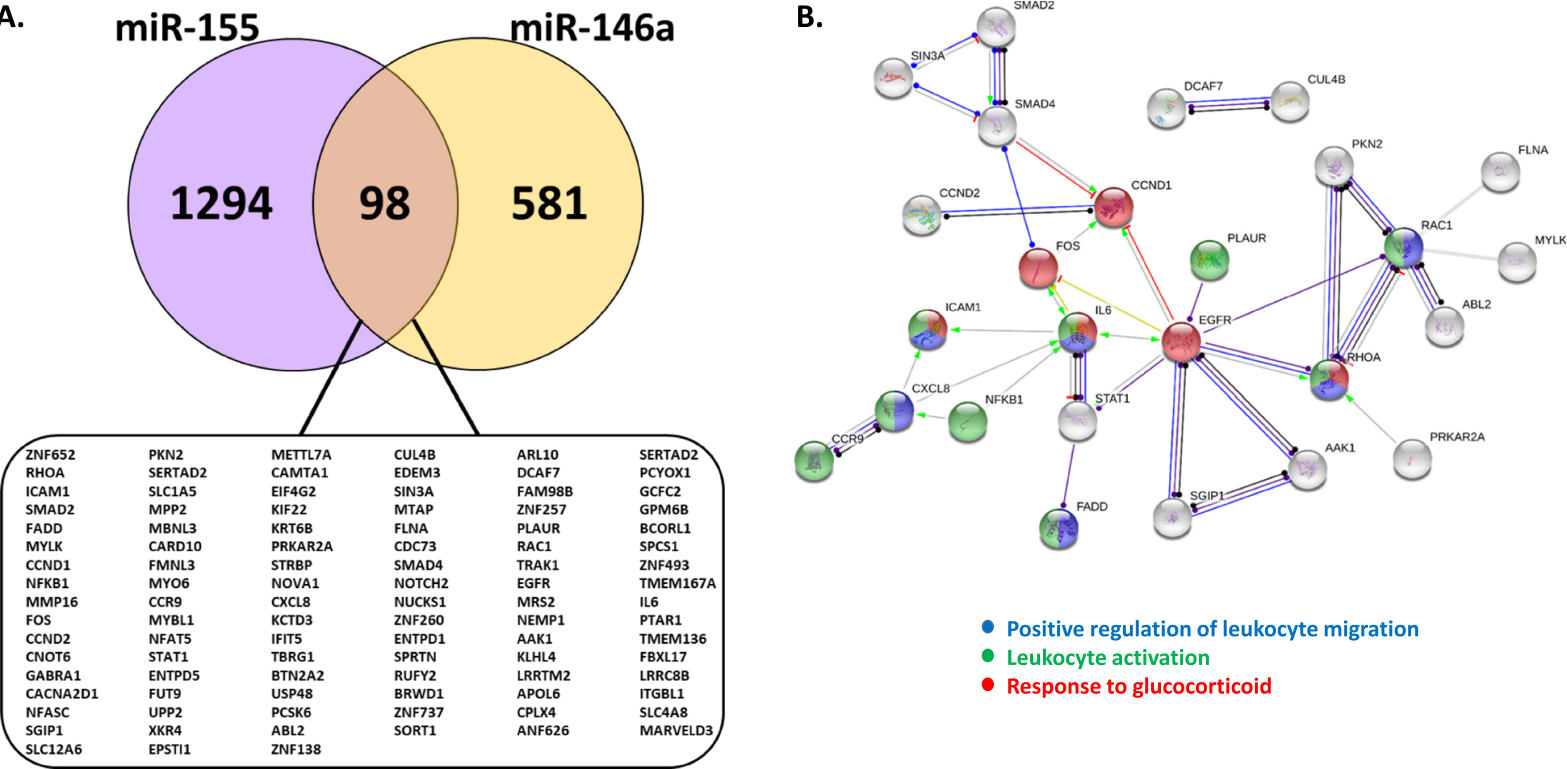
miRNAs correlated to increased inhaled corticosteroid usage are enriched in immune associated genes. As miR-155 and miR-146a both positively correlated to inhaled corticosteroid usage, common gene targets were identified and pathway analysis was performed. (**a)** Predicted and verified targets for miR-155 and miR-146a were compared. Common gene targets are shown in the box. (**b)** Shows all common connected gene targets. The protein-protein interaction value for the analysis was significant (*p* = 0.00336). Targets identified as being involved in immune processes are colored: response to glucocorticoid (red), positive regulation of leukocyte migration (blue) and leukocyte activation (green). Pathways were generated using String [39]

### Leukocyte regulation and glucocorticoid response are enriched in genes targeted by miRNAs increased under inhaled corticosteroids usage

As miRNAs can act both in an intracellular and extracellular manner, we wondered what role these circulating miRNAs may elicit on target cells. Thus, we compiled a list of shared predicted gene targets between the two miRNAs increased upon ICS usage, miR-155 and miR-146a, and pathway analysis was performed. Ninety-eight common genes were determined and pathways were generated (Fig. 5a&b). Furthermore, twenty-six nodes were connected and showed a significant degree of protein-protein interactions (*p* < 0.00336; Fig. 5b). GO analysis for biological processes revealed that some of the most enriched pathways with these particular genes included cellular response to interleukin (IL)-6 and IL-6 signalling, positive regulation of leukocyte migration and response to glucocorticoid (Supp Table 2). Similarly, reactome pathways were also enriched in various interleukin signalling pathways (Supp Table 3).

## Discussion

In this study we have shown that different asthma subgroups exhibited altered expression in circulating miRNAs. We found that miR-155 and miR-146a expression in AAs was significantly increased when the examined subjects were using ICS. Furthermore, we found that miR-223 and miR-374a showed significant expression changes in NAAs when blood eosinophil numbers were taken into account. Additionally, several miRNAs correlated to lung function parameters, circulating immune cells, atopy and ICS usage. Finally, we found that miRNAs with increased expression upon ICS usage, miR-155 and miR-146a, shared target genes involved in leukocyte regulation and response to glucocorticoids. Our results suggest that combinations of circulating miRNAs can aide in defining patient groups in asthma.

The finding that miR-155 is increased in serum samples from people with allergic asthma compared to non-allergic asthmatics and healthy individuals is interesting. We recently showed miR-155 to be a critical regulator of type 2 innate lymphoid cells in murine models of allergic airway inflammation [25] and to be differentially expressed in the airways of allergic asthmatic individuals compared to healthy controls [24]. A recent study in CD4^+^ T-cells demonstrated that miR-155 expression was altered in response to allergic stimuli or glucocorticoid treatment in a set of dust-mite allergic rhinitis, healthy, and asthmatic subjects [32]. Unlike our data, Daniel et al. observed that miR-155 expression was increased in allergic subjects and was significantly down-regulated upon addition of dexamethasone. Some of the disparity may occur from the sample type used as we used serum from whole blood and the previous study examined isolated CD4^+^ T-cells. Furthermore, our subjects were examined at baseline whereas Daniel et al. stimulated cells with the dust mite extract allergen [32]. miR-155 is likely one of the most studied miRNAs to date, is implicated in a variety of diseases and prevalently studied in asthma [24–26]. As we do not know the cellular origin of our examined circulating miRNAs, we cannot currently identify which cells are responsible for the increased expression observed in ICS treated individuals.

Glucocorticoids (GCs) are often the first and most common line of treatment for most asthmatics and work by dampening or inhibiting type 2 immune responses [40]. Although studies have noted the usage of ICS in their subjects, few have looked at ICS as a parameter for miRNA expression [10–12, 14, 41]. In our cohort, we observed changes to miRNA expression in AAs with or without ICS usage, but NAA subjects regardless of ICS status exhibited miRNA expression levels similar to healthy subjects. This finding again highlights the differences in AA and NAA subgroups, suggesting a different underlying mechanism for the disease. As in our study, another recent study of childhood asthma also found miR-146a to be increased in children on ICS, thus suggesting that this miRNA may be useful even as an early biomarker for asthma [42]. Although ICS works very well for most atopic subjects with type 2 asthma, there are other subgroups of asthma that are non-atopic and have varying levels of type 2 response (high or low) and there are subjects that are steroid resistant [2]. For these individuals, GCs often do not alleviate the symptoms of the disease, which may result in costly trips to the hospital in case of exacerbation. In most cases, the molecular mechanisms controlling these forms of asthma are unknown. Therefore, it is of special interest to identify and define these asthma subgroups from not only a clinical perspective, but to elucidate the molecular pathogenesis of the disease, thus, defining asthma endotypes.

In contrast to the AA group, the miRNA expression in the NAA group did not change in response to ICS usage. However, miR-223 expression was increased in individuals with high circulating eosinophil levels and significantly decreased in individuals with low circulating eosinophil levels as compared to healthy individuals. When examining clinical parameters in our study, we found the strongest significant correlation of miR-223 with eosinophils as well as ECP, suggesting that this miRNA may have a role in eosinophil development in asthmatic subjects. miR-223 is of particular interest in a type 2 inflammation driven-disease such as asthma where increased eosinophil numbers are often used as a clinical biomarker. Interestingly, Lu et al. observed miR-223, one of the most upregulated miRNAs found in eosinophilic esophagitis, was strongly correlated to eosinophil levels in the esophagus [43]. The group later reported that deficiency of miR-223 led to an increase in eosinophil progenitors in bone marrow of mice [44]. Although the role of miR-223 in circulating eosinophils is not known, perhaps the increased miR-223 expression we observe in NAAs with high eosinophil counts could lead to lower levels of eosinophil progenitors in the blood. Conversely, the decrease in miR-223 expression even below that of healthy individuals in NAA subjects with low levels of eosinophils (< 0.1 × 10^9^ cells /L), may suggest an imbalance in eosinophilopoesis, potentially leading to the manifestation of their asthma subtype. However, in order to determine the relationship and importance of miR-223 and eosinophils, miR-223 must be confirmed to be expressed in human eosinophils as we have examined miRNA expression only in cell-free serum. Furthermore, we observed an interesting opposing relationship between miR-146a and miR-223. In our study, these miRNAs exhibited inverse levels of correlations to clinical parameters (Fig. 4d-f; e.g., ECP, eosinophils, FVC) and exhibited a significant negative correlation to one another (Supp Fig. 2), perhaps suggesting different cellular origins of these miRNAs. Additionally, Panganiban et al. found that both miR-146a and miR-223 expression were increased in plasma from asthma subjects as opposed to healthy or allergic rhinitis individuals [14]. Even in murine models of asthma treated with ovalbumin or house dust mite, miR-146a and miR-223 were differentially regulated in plasma [27]. We also observed that miR-146a and miR-223 showed altered expression patterns between asthma and healthy individuals, but also between our asthma subgroups, with miR-146a exhibiting expression changes in AAs, whereas miR-223 levels differed in NAAs. With these previous studies and our own, both miR-146a and miR-223 appear to be robust miRNAs in blood, able to be identified in both plasma and serum and act as distinguishing markers for asthma subgroups.

miR-374a is a less studied miRNA which has been identified in several screens, but its function still remains elusive [12, 14, 41]. We found that miR-374a exhibits different expression patterns in AA and NAA asthmatics (Fig. 2c&g and Fig. 3d). We observed that in NAAs, miR-374a appeared to be influenced by circulating eosinophil level rather than ICS status (Fig. 3d), whereas in the AAs, miR-374a clearly showed a strong decrease in expression in ICS naïve individuals (Fig. 2c&g). Further studies to determine the cellular origin of miR-374a and potential gene targets may help to elucidate the functional role this miRNA plays in asthma. In both our study and Kho et al., it was observed that miR-374a correlated to lung function, specifically FEV1/FVC [12]. Interestingly, in the childhood serum cohort examined by Kho et al., the correlation of FEV1/FVC to miR-374a expression was positive, whereas we observed a negative correlation (Fig. 4d). We did, however, find a positive correlation with miR-374a expression and age. Perhaps this discrepancy in lung function correlation lies in the age of the cohort as we examined an adult cohort (average age 48 years) and Kho and colleagues worked with a childhood asthma cohort [12]. In order to determine if age and miR-374a expression are associated with lung function, future longitudinal studies would be required.

Through the use of non-invasive biomarkers, we can start to tease apart potential epigenetic markers of interest, predict and study their targeted genes and pathways and propose and develop potentially life altering treatments for numerous individuals. We performed a pathway analysis on common targets of miR-155 and miR-146a, which were found in this study to be increased specifically in AA+ICS individuals (Fig. 5), to gain insight into their potential function. We observed that of the ninety-eight common genes, twenty-six formed a network and the majority of those genes were enriched in biological processes such as leukocyte regulation and response to GCs. As the purpose of ICS are to dampen the type 2 response, it may be that ICS cause an increase in post-transcriptional regulators, such as miR-155 and miR-146a. These regulators may then be involved in the negative regulation of cells involved in type 2 response, thus, alleviating asthma symptoms. We also found an enrichment in genes associated with IL-6 signaling (Supp Table 1 ). IL-6 signaling has been previously suggested as a biomarker for asthma [45–47] and circulating levels of IL-6 has been shown to be increased in individuals with asthma [47] and has been associated with high dose ICS in adult onset asthma [45]. Due to the complexity of IL-6 signaling, it was outside the scope of the current study, but would be a potentially interesting avenue for future research.

Although we have identified miRNAs that may be useful as potential future biomarkers, one limitation of our study is that we have a relatively small number of subjects. Through multivariate logistical regression analysis, we determined the potential biomarker quality of the six candidate miRNAs (Supp Fig. 3). Although this model appears to be in agreement with our previous statistical analysis, the small subject size (*n* = 92) and number of variables (*n* = 11) is likely an overestimation of the predictive power of the given miRNAs. Our data suggests that levels of circulating blood eosinophils may influence the relative expression of certain miRNAs, such as miR-223 and miR-374a. Unfortunately, as we have looked at only serum, we cannot be sure of the origin of these miRNAs. In the future, it would be interesting to determine if indeed the aforementioned miRNAs are more abundant in eosinophils compared to others miRNAs that we have examined. Furthermore, in this study we have chosen only six miRNAs to examine based on their previously reported roles in human asthma and inflammation or murine models of asthma [4, 20–33]. In order to gain a better understanding of all the alterations in miRNAs among asthma subgroups, it would be beneficial to use a screening technique such as microarray, Nanostring analysis or RNA-sequencing. Indeed, Rodrigo-Muñoz et al. performed RNA-sequencing in eosinophils and identified several miRNAs other than those which we have examined that were specific in distinguishing asthmatic individuals [48] .

## Conclusion

Our study shows that several miRNAs found in the serum correlate to clinically relevant parameters and are significantly and differentially expressed not only between healthy individuals and asthmatics, but also between defined asthma subgroups. Based on ICS status or circulating eosinophil numbers, we were able to identify miRNAs whose expression aides in distinguishing between asthma subgroups. These observed differences may in the future be useful in predicting or better defining asthma subtypes, but these results need to be recapitulated in a larger cohort. Furthermore, pathway analysis of potential gene targets suggest that these miRNAs may be involved in immunologically relevant processes such as suppression of leukocyte activation and IL-6 signaling. Although the origin of these miRNAs is yet unknown, they serve as a basis for non-invasive potential biomarkers to better classify asthma subgroups.

## Supplementary information

**Additional file 1: Supp Fig. 1.** miR-126 expression is unaffected by asthma subtype, eosinophil level or ICS usage. **Supp Fig. 2.** Correlations of examined serum miRNA expression in healthy and asthma subjects. **Supp Fig. 3.** Multiple logistic regression to determine miRNA strength in distinguishing between asthma groups. **Supp Table 1.** Biological Processes enriched in miR-155 and miR-146a gene targets. **Supp Table2.** Reactome Pathways enriched in miR-155 and miR-146a gene targets. **Supp Table 3.** KEGG Pathways enriched in miR-155 and miR-146a gene targets.

## Data Availability

Please contact the authors for data requests.

## Abbreviations

AA: Allergic asthmatic
ECP: Eosinophil cationic protein
FEV1: Forced expiratory volume in 1 s
FVC: Forced volume capacity
GO: Gene ontology
ICS: Inhaled corticosteroids
miRNA/miR: microRNA
NAA: Non-allergic asthmatic
WSAS: West Sweden Asthma Study
