# Supplementary material for: Circulating microRNAs correlate to clinical parameters in individuals with allergic and non-allergic asthma
Source: Respir Res. 2020 May 7;21:107. doi: 10.1186/s12931-020-01351-x (PMC7203878; doi:10.1186/s12931-020-01351-x)
Supplement: Supplementary file 1 — Additional file 1: Supp Fig. 1. miR-126 expression is unaffected by asthma subtype, eosinophil level or ICS usage. Supp Fig. 2. Correlations of examined serum miRNA expression in healthy and asthma subjects. Supp Fig. 3. Multiple logistic regression to determine miRNA strength in distinguishing between asthma groups. Supp Table 1. Biological Processes enriched in miR-155 and miR-146a gene targets. Supp Table2. Reactome Pathways enriched in miR-155 and miR-146a gene targets. Supp Table 3. KEGG Pathways enriched in miR-155 and miR-146a gene targets. [file 12931_2020_1351_MOESM1_ESM.docx]

**Supplementary Materials**

**Circulating microRNAs correlate to clinical parameters in individuals with allergic and non-allergic asthma**

Running title: Serum miRNAs are altered in asthma subgroups

Julie Weidner^1^, Linda Ekerljung^1^, Carina Malmhäll^1^, Nicolae Miron^2^, Madeleine Rådinger^1^

^1^ Krefting Research Centre, Sahlgrenska Academy, University of Gothenburg, Sweden

^2^ Clinical Immunology, Sahlgrenska University Hospital, Gothenburg, Sweden

Corresponding author: [madeleine.radinger@gu.se](mailto:madeleine.radinger@gu.se)

**Supplementary Materials:**

Supp fig 1

**Supp Figure 1: miR-126 expression is unaffected by asthma subtype, eosinophil level or ICS usage**

Individuals with asthma (allergic-AA; non-allergic-NAA) were divided based on circulating eosinophil cell level where High Eos are >0.4 x10^9 cells/L and healthy subjects and Low Eos individuals had <0.1x10^9 cells/L and inhaled corticosteroid (ICS) usage. Each point represents one individual. The line indicates the median.


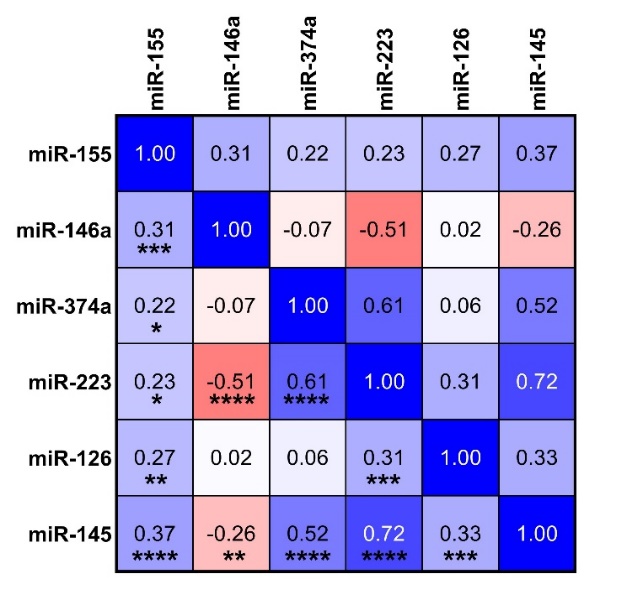

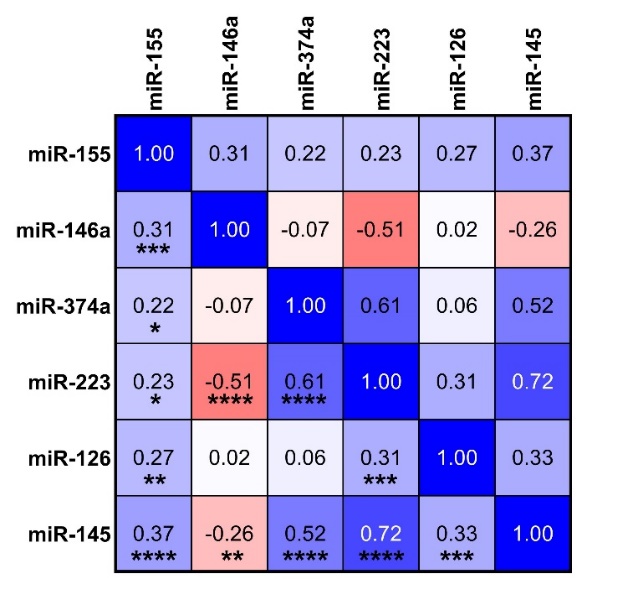

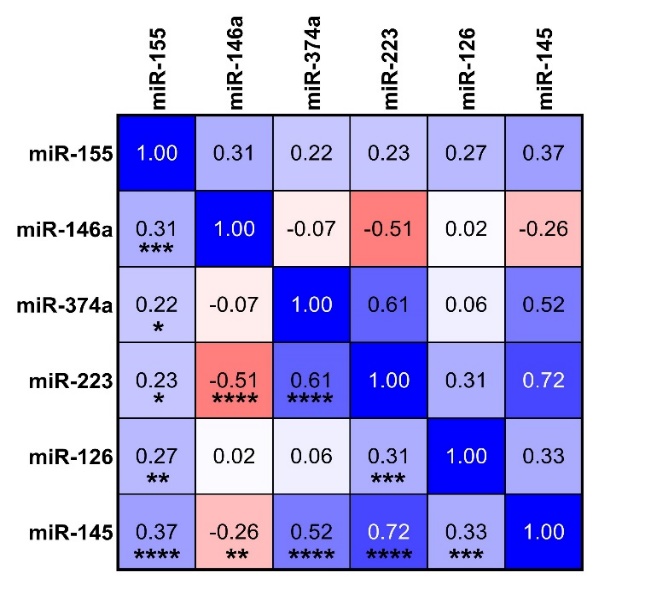

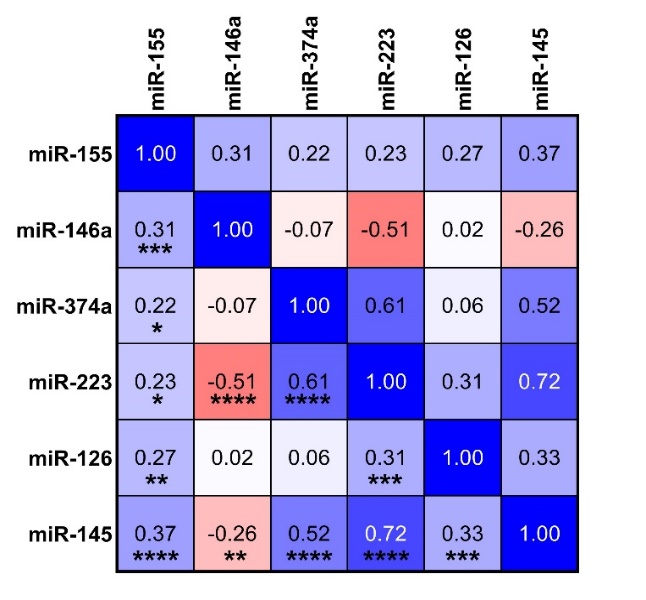

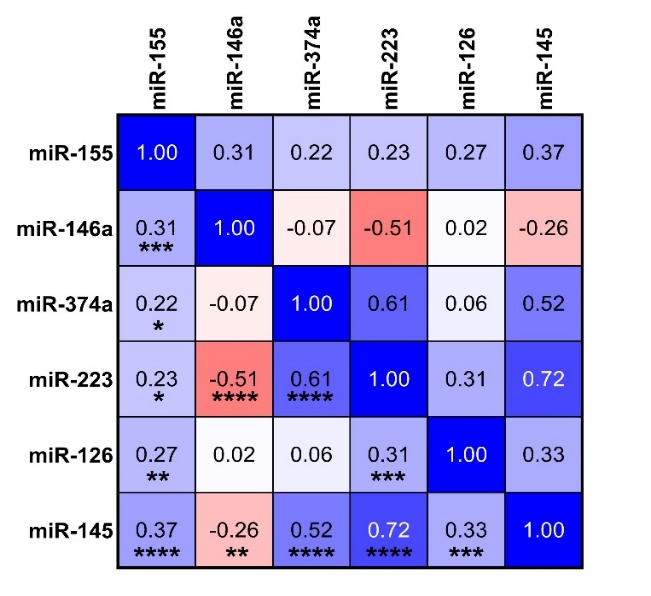

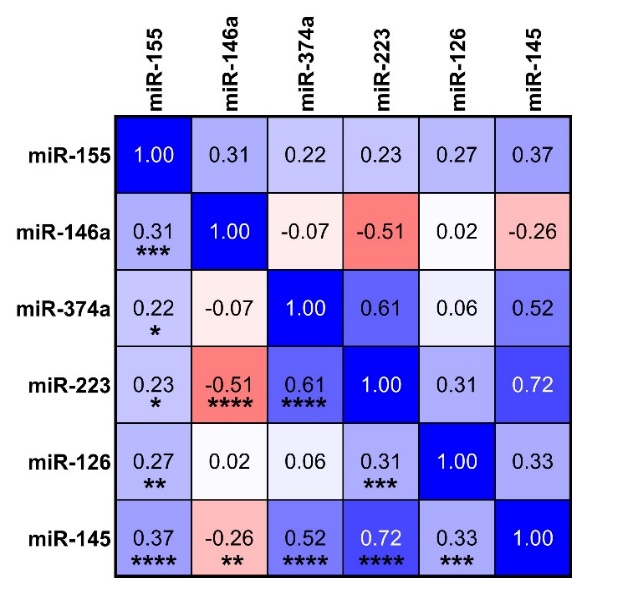

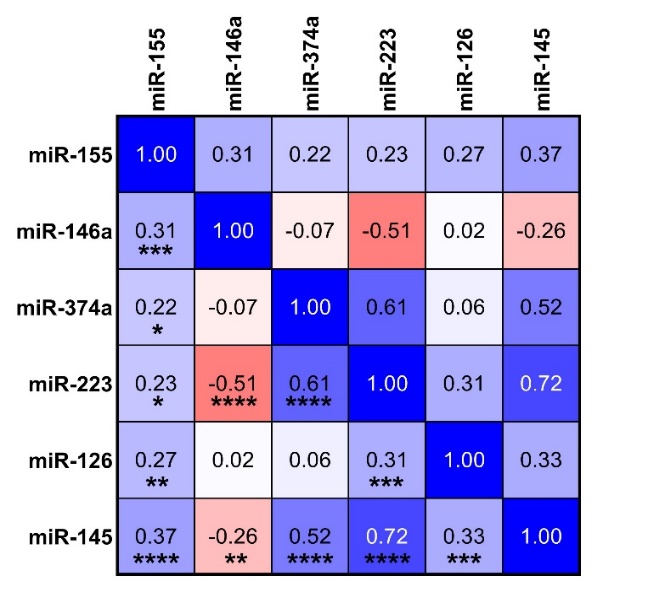


Supp fig 2

**Supp Figure 2: Correlations of examined serum miRNA expression and clinical parameters in healthy and asthma subjects**

Spearman correlations were performed between circulating miRNA expression. Shown is the cut out and enlarged section from from the larger correlation matrix in Fig 4. The boxes contain the correlation coefficients (r) for each pair and significant correlations (*) if present. p<0.05=*; p<0.01=**; p<0.001=***; p<0.0001=****


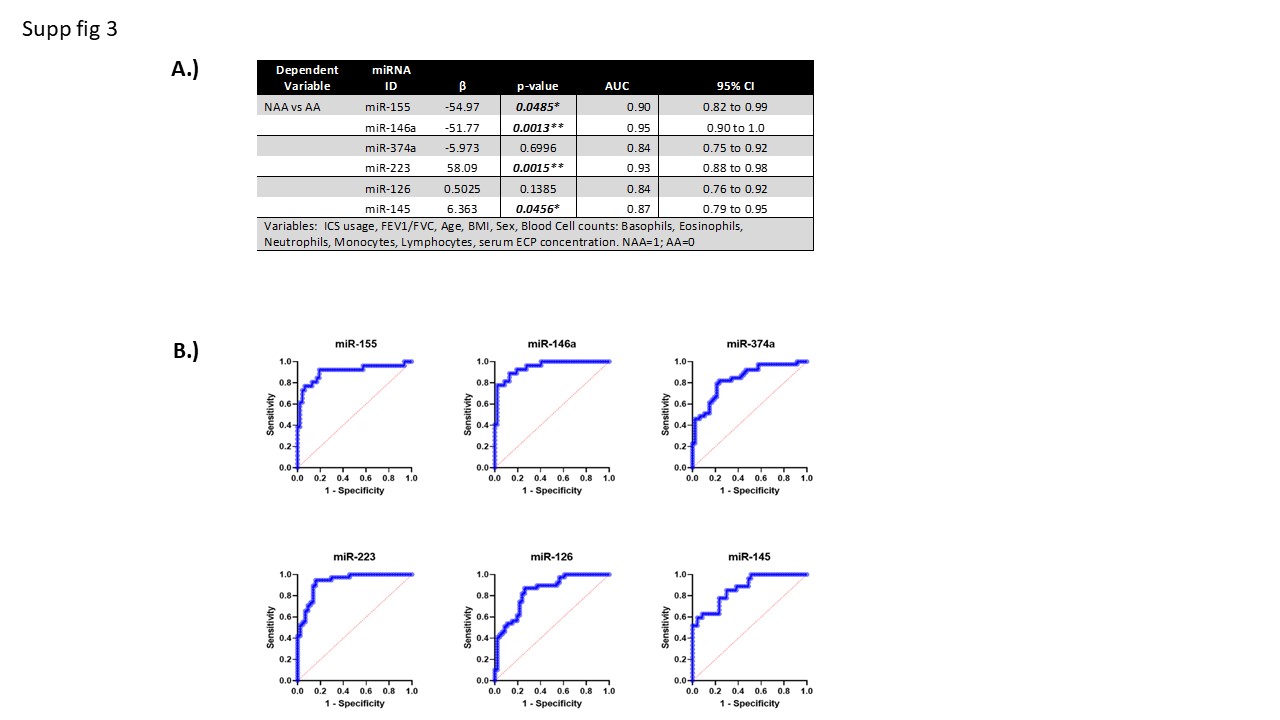


Supp fig 3

**Supp Figure 3: Multiple logistic regression to determine miRNA strength in distinguishing between asthma groups**

**A.)** Table indicating the parameter estimate (β) for each miRNA as well as the area under the curve (AUC) with 95% confidence interval (CI) for each candidate miRNA. A significant p-value is highlighted in **bold italics.** Variables used in the analysis are listed under the table. p<0.05=*; p<0.01=**

**B.)** Receiver operating curves for each individual miRNA from table **(A)**

Supp Table 1

**Supp Table 1: Biological Processes enriched in miR-155 and miR-146a gene targets**

Listed are the top 50 significant biological processes as identified using String^39^. The number of target genes common to the process and the false discovery rate is shown.

Supp Table 2


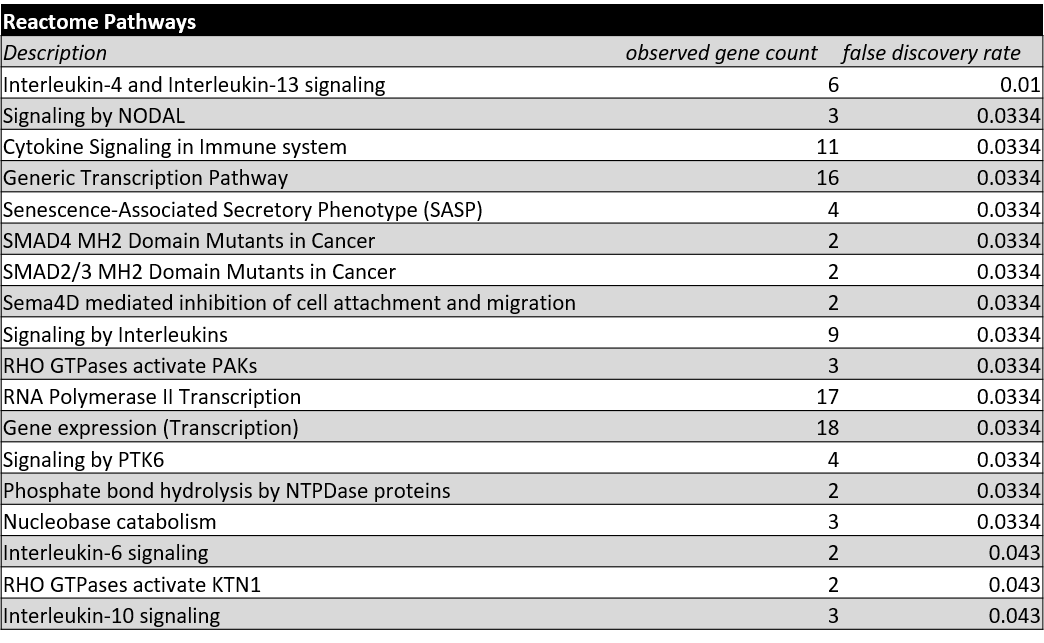


**Supp Table 2: Reactome Pathways enriched in miR-155 and miR-146a gene targets**

Listed are the significant reactome pathways as identified using String^39^. The number of target genes common to the process and the false discovery rate is shown.

**Supp Table 3: KEGG Pathways enriched in miR-155 and miR-146a gene targets**

Supp Table 3

Listed are the significant KEGG pathways with at least 3 common target genes as identified using String^39^. The number of target genes common to the process and the false discovery rate is shown.
